# Annual acknowledgement of manuscript reviewers

**DOI:** 10.1186/1550-2783-10-12

**Published:** 2013-03-20

**Authors:** Jose Antonio, Douglas Kalman, Richard Kreider

**Affiliations:** 1 Exercise and Sports Science, Nova Southeastern University, Davie, FL, 33314, USA; 2 Miami Research Associates, Endocrinology & Nutrition Department, 6141 Sunset Drive - Suite 301, Miami, FL, 33143, USA; 3 Exercise & Sport Nutrition Lab, Department of Health & Kinesiology, Texas A&M University, College Station, TX, USA

## Abstract

**Contributing reviewers:**

The editors of Journal of the International Society of Sports Nutrition would like to thank all our reviewers who have contributed to the journal in Volume 9 (2012).

## 

Michelle Adams

United States of America

Ajmol Ali

New Zealand

Dawn Anderson

United States of America

Hans Joachim Appell

Germany

Alan Aragon

United States of America

Giannis Arnaoutis

Greece

Eldon Askew

United States of America

Mohammad Ali Azarbayjani

Iran

Norbert Bachl

Austria

Jenna Becker

United States of America

Sandeep Bhale

United States of America

Douglas Bibus

United States of America

Francois Blachier

France

Andrew Bosch

South Africa

Luiz-Claudio Cameron

Brazil

Bill Campbell

United States of America

Darren Candow

Canada

Ana Castilla

United States of America

Eric Claassen

Netherlands

Gillian Cowen

Australia

Kirk Cureton

United States of America

Claudia da Luz

Brazil

Maeli Dal Pai

Brazil

Lara Dugas

United States of America

Charles Dumke

United States of America

Inna Dumova

United States of America

Christopher Dunbar

United States of America

Conrad Earnest

United Kingdom

Joan Eckerson

United States of America

Mohamed Elloumi

Tunisia

Matt Fitzgerald

United States of America

Donovan Fogt

United States of America

Emerson Franchini

Brazil

Karl Friedl

United States of America

Yukio Fujita

Japan

Moacir Godoy

Brazil

Marcela Gonzalez-Gross

Spain

Mike Greenwood

United States of America

Bruno Gualano

Brazil

Marios Hadjicharalambous

Cyprus

Matthew Harber

United States of America

John Hawley

Australia

Stephen Heinrichs

United States of America

Jay Hoffman

United States of America

Geoffrey Hudson

United States of America

Mikel Izquierdo

Spain

Patrick Jacobs

United States of America

Ralf Jäger

United States of America

Lynne Kammer

United States of America

Chad Kerksick

United States of America

Jeong-Su Kim

United States of America

Michael Kingsley

United Kingdom

Paul La Bounty

United States of America

Antonio Lancha

Brazil

Joan Lappe

United States of America

Hubert Lee

United States of America

Anna Lepeley

United States of America

Shiming Li

United States of America

Hector Lopez

United States of America

Ryan Lowery

United States of America

Shannan Lynch

United States of America

Gerald Mangine

United States of America

James McClung

United States of America

Brendon McDermott

United States of America

Andrew McKune

South Africa

Andrea McNeilly

United Kingdom

Jamie McPhee

United Kingdom

Vazgen Minasian

Iran

Daniel Moran

Israel

Elizabeth Nagle

United States of America

Anthony Nakazawa

United States of America

Angelica Neris

United States of America

Layne Norton

United States of America

Michael Ormsbee

United States of America

Hyon Park

South Korea

Mário Paschoal

Brazil

Tavis Piattoly

United States of America

Nicholas Ratamess

United States of America

John Rathmacher

United States of America

Anthony Ricci

United States of America

Tania Rivera

United States of America

Michael Roberts

United States of America

Scott Robinson

United Kingdom

Hamilton Roschel

Brazil

Edward Ryan

United States of America

Craig Sale

United Kingdom

Stephen Schmitz

United States of America

Brad Schoenfeld

United States of America

Christopher Scott

United States of America

James Scott

United States of America

Richard Seip

United States of America

Piero Sestili

Italy

Andrew Shao

United States of America

Tobin Silver

United States of America

Dustin Slikva

United States of America

Abbie Smith

United States of America

Jeffrey Stout

United States of America

Tetsuya Takamata

Japan

Lemuel W Taylor

United States of America

Toni Torres-McGehee

United States of America

Filomena Valim

United States of America

Xuewen Wang

United States of America

Louise Weschler

United States of America

Urban Wiklund

Sweden

Colin Wilborn

United States of America

Darryn Willoughby

United States of America

Jacob Wilson

United States of America

Gabriel Wilson

United States of America

Kuan Zhang

China

Tim Ziegenfuss

United States of America

